# Supplementary material for: The interactome of intact mitochondria by cross-linking mass spectrometry provides evidence for coexisting respiratory supercomplexes
Source: Mol Cell Proteomics. 2017 Dec 8;17(2):216–32. doi: 10.1074/mcp.RA117.000470 (PMC5795388; doi:10.1074/mcp.RA117.000470)
Supplement: Supplemental Data [file supp_RA117.000470_133922_0_supp_23978_fzffwf.zip › spectra_annotation/mito_spectra_annotation/315-1-14-1-10-1.pdf]

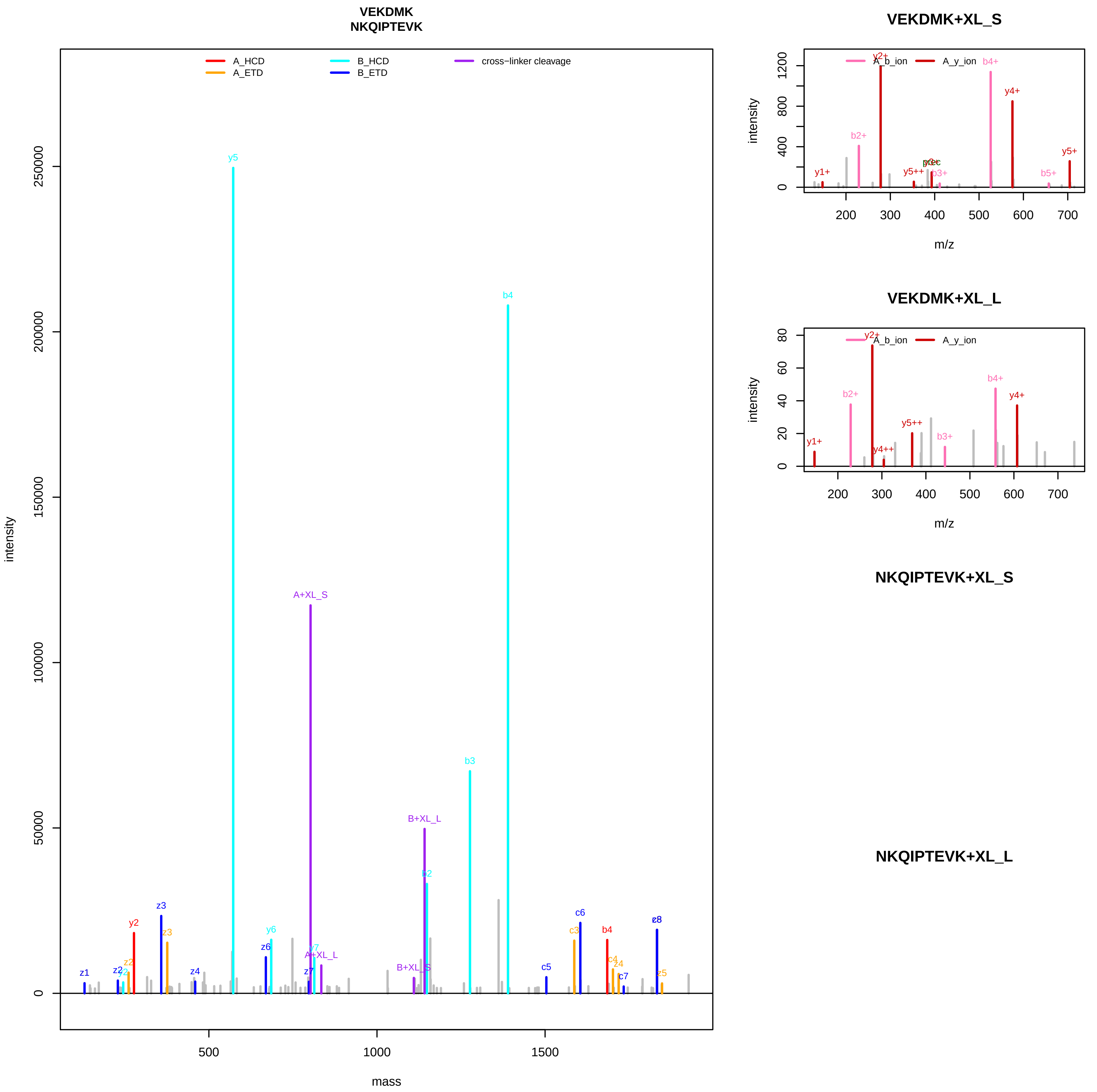
VEKDMK
NKQIPTEVK
A_HCD
A_ETD
B_HCD
B_ETD
cross−linker cleavage
intensity
mass
0
50000
100000
150000
200000
250000
500
1000
1500
y5
b4
A+XL_S
B+XL_L
b3
b2
y6
z6
z3
y2
z3
z2
z1
z4
y7
A+XL_L
z7
B+XL_S
c5
c3
c6
b4
c4
z4
c7
z8
z5
VEKDMK+XL_S
A_b_ion
A_y_ion
m/z
y2+
b4+
y4+
b2+
y5+
y1+
y5++
b3+
b5+
VEKDMK+XL_L
y2+
b2+
b4+
y4+
y5++
b3+
y4++
y1+
NKQIPTEVK+XL_S
NKQIPTEVK+XL_L
